# Beneficial effects of cocoa on lipid peroxidation and inflammatory markers in type 2 diabetic patients and investigation of probable interactions of cocoa active ingredients with prostaglandin synthase-2 (PTGS-2/COX-2) using virtual analysis

**DOI:** 10.1186/2251-6581-13-30

**Published:** 2014-02-04

**Authors:** Nayereh Parsaeyan, Hassan Mozaffari-Khosravi, Abdorrahim Absalan, Mohammad Reza Mozayan

**Affiliations:** 1 Department of Biochemistry, Faculty of Medicine, Shahid Sadoughi University of Medical Sciences, Yazd, Iran; 2 Department of Nutrition, Faculty of Health, Shahid Sadoughi University of Medical Sciences, Yazd, Iran; 3 Department of Clinical Biochemistry, Faculty of Medicical Sciences, Tarbiat Modares University, Tehran, Iran; 4 Department of English Language, Shahid Sadoughi University of Medical Science, Yazd, Iran

**Keywords:** Cocoa, Diabetes type 2, Lipid Peroxidation, Inflammatory Markers, Docking

## Abstract

**Background and aim:**

Altered glucose metabolism, oxidative stress, lipid levels and inflammatory markers are important risk factors in diabetes, cardiovascular, and many other diseases. Cocoa has been shown to exert antioxidant and anti-inflammatory effects. The aim of this study is twofold: to assess the effect of Cocoa on the lipid profile and peroxidation in addition to the inflammatory markers in type 2 diabetic patients, and to represent a virtual model of probable action mechanism of observed clinical effects of Cocoa consumption using in silico analysis and bioinformatics data.

**Methods:**

One hundred subjects with type 2 diabetes were included in a randomized clinical control trial. Fifty treatment subjects received 10 grams cocoa powder and 10 grams milk powder dissolved in 250 ml of boiling water, and the other fifty control subjects received only 10 grams milk powder dissolved in 250 ml boiling water. Both groups were on the mentioned regimen twice daily for 6 weeks. Blood samples were obtained prior to Cocoa consumption and 6 weeks after intervention. Serum lipids and lipoproteins profile, malondialdehyde and inflammatory markers including tumor necrosis factor-α (TNF-α), interleukin-6 (IL-6) and high sensitive C-reactive protein (hs-CRP) were measured. For statistical analysis two independent and paired samples t-test and linear regression were used. Bioinformatics and virtual analysis were performed using string data base and Molegro virtual software.

**Results:**

Cocoa consumption lowered blood cholesterol,triglyceride, LDL-cholesterol, and TNF-α, hs-CRP, IL-6 significantly (P < 0.01). The results showed that the levels of HDL-cholesterol decreased significantly (P < 0.05) but Cocoa inhibited lipid peroxidation in treatment group than control group (P < 0.0001). Virtual analysis showed that the most frequent Cocoa ingredients, (+)-Catechin and (−)-Epicatechin, can dock to the enzyme COX-2.

**Conclusion:**

These data support the beneficial effect of Cocoa on the lipid peroxidation prevention and inflammatory markers in type 2 diabetic patients. Cocoa ingredients block the Cox-2 activation and reduce inflammatory prostanoids synthesis according to virtual analysis.

## Introduction

Insulin resistance and diabetes contributes to metabolic disturbances [1]. Inflammation which occurs during immunological responses can also be regarded as a complication resulting from a changed metabolism [2,3]. Moreover, the main risk factor for diabetic patient’s death is cardiovascular disease (CVD). Although CVD-caused mortality has decreased since the past decades, it still accounts for higher than 40% of the total mortality rate [4]. CVD is widely asserted to be associated with elevated oxidative stress [5]. Lipid peroxidation is the most commonly assessed process in oxidative stress research. There are various plasma markers of lipid peroxidation including Malondialdehyde, lipid hydroperoxides, conjugated dienes, oxidation resistance assay (lag time), oxysterols and F2α-isoprostanes [6]. Historically, the most common method for measuring lipid peroxidation has been the thiobarbituric acid reactive substances (TBARS) assay to quantify malondialdehyde [6]. Although increased risk of coronary artery disease (CAD) has contributed to hyperglycemia, dyslipidemia and thrombotic state, recent studies have come to focus on inflammatory markers [7]. Markers of chronic low-grade inflammation, such as tumor necrosis factor-α (TNF-α), Interleukin-6 (IL-6), and high sensitive- C-reactive protein (hs-CRP) have been shown to predict the risk of developing type 2 diabetes and cardiovascular disease. Moreover, they are conceived to be directly involved in the pathogenesis of such chronic diseases [8]. TNF-α and IL-6 belong to cytokines which are predominantly secreted from adipose tissues while hs-CRP is the principal downstream mediator of the acute phase response and is secreted by the liver in response to TNF-α and IL-6 [9].

Nutrition plays a key role in the prevention of many chronic diseases including CVD, cancers, diabetes, and inflammatory diseases [10]. Cocoa is known to be rich in polyphenols, such as catechin, epicatechin, procyanidin B2 (dimer), procyanidin C1 (trimer), cinnamtannin A2 (tetramer), and other oligomer procyanidins [11]. Studies on healthy human subjects have indicated decreased levels of serum LDL-cholesterol and increased levels of HDL-cholesterol. Furthermore, resistance of LDL-cholesterol to oxidation following the intake of dairy Cocoa powder has been reported previously [12,13]. In vitro studies have suggested that Cocoa procyanidins and phenolic metabolites can also modify intracellular signal transduction pathways thereby modulating the synthesis of inflammatory cytokine such as IL-6 [14].

Prostaglandin synthase-2 (PTGS-2) which is mostly defined as cyclooxygenase-2 (COX-2) enzyme, is responsible for the production of the prostaglandins following inflammation. Activation of PTGS-2 results in the rate limiting step in inflammatory prostaglandins production [15] and it is induced after activation of cytokines, mitogens and endotoxins. Its transcription is potentially activated following inflammation induction in inflammatory cells [16]. Computational biology and bioinformatics studies are helpful to explore probable interactions of biomolecules with chemicals such as herbal active ingredients at least in the case of the absence of real models, such as pure in vitro models.

Phenolic ingredients of Cocoa exert potent antioxidant properties in-vitro. Flavanols and flavonoids founded in tea and Cocoa shown to have anti-oxidant and anti-inflammatory effects such as platelet activation inhibition [17]. Different actions have been reported for Cocoa ingredients, ie., slowing down LDL-oxidation [18,19] and reducting inflammatory cytokines synthesis [20] modulation of TNF-α secretion from mononuclear blood cells [21]. However, exact molecular mechanism of such actions is not clearly identified. The aim of this study is to evaluate the effect of 6-week regimen of cocoa consumption on lipid profile,lipoproteins peroxidation and inflammatory markers including TNF-α, IL-6 and hs-CRP in a randomized controlled clinical trial. Moreover, using an in silico simulation, probable interactions of PTGS-2/COX-2 with two most frequent phytochemical ingredients of Cocoa, (+)-Catechin and (−)-Epicatechin are evaluated. This contributes to address whether the active ingredient of Cocoa target inflammation-inducer enzymes is COX-2 and whether this is the probable mechanism of Cocoa ingredient anti-inflammatory action.

## Materials and methods

One hundred subjects affected with type 2 diabetes participated in the study. Patients were from the data base of Yazd Diabetes research center who were selected by a simple sampling method. The eligibility criteria were as follows: age between 40–70 years; total cholesterol above 240 mg/dl; triglyceride higher than 200 mg/dl; no regular consumption of supplements affecting lipid metabolism; no extreme exercise habits; and no history of any anti-hyperlipidemia drugs usage. Study participants were informed about the research status and only those who approved and verified the consent form were included. Moreover, the study situations and ethical confirmation were determined by the ethic committee of Shahid Sadoughi University of Medical Sciences. The trial has been registered in Iranian Registry of Cilinical Trials at http://www.irct.ir with the following identification: IRCT201101265697NI. Virtual analysis was performed using Molegro Virtual Docker Software Ver. 4.2. Furthermore, interactive network was obtained from STRING data base. Protein data bank was also another data base to explore the properties and characteristics of Prostaglandin Synthase-2 (PTGS-2/COX-2) enzyme. Cheminformatics structures of active ingredients of Cocoa, (+)-Catechin and (−)-Epicatechin, were obtained from Zinc data base as well.

### Experimental design

One hundred type 2 diabetic patients were randomly divided into two categories of 50 individuals. Fifty patients were assigned to the treatment group (TG) and instructed to consume 10 grams cocoa powder added to 10 grams dried milk powder both dissolved in 250 ml boiling water. The regimen, daily twice, continued for 6 weeks. The control group (CG) was also instructed to use only 10 grams of milk powder dissolved in 250 ml of boiling water with the same regimen period as the other group (twice daily for 2 weeks). The regimen would have to be applied before noon and during afternoon. To make sure that the specified regimen foods would be consumed by all the subjects, arrangements were made to supply home deliveries of packaged powder to each subject 1 week prior to consumption until the termination of the study. Furthermore, clear guidance was represented based on the need of the subjects to maintain their routine diets for breakfast, lunch, drinks, incidental foods and therapeutic drugs regimen. In addition, in order to check that the normal diets would be maintained, the subjects’ complete dietary records throughout the study were kept. The subjects were also advised to avoid taking any supplements and all other cocoa products and to lead their usual life style throughout the study. Blood samples were then obtained from all volunteers prior to Cocoa consumption and 6 weeks after intervention. Blood cholesterol, triglycerides and HDL-cholesterol (HDL-C) were measured by enzymatic colorimetric kit methods (Pars Azmun Co.) according to package insert instructions. Low density lipoprotein cholesterol (LDL-C) was calculated using Friedewald formula [22].

Malondialdehyde, ie end product of lipid peroxidation, levels were measured by thiobarbituric acid reactive substances (TBARS) Method [23]. Three inflammatory markers such as hs-CRP, IL6 and TNF-α serum levels were also measured by a sandwich ELISA methods (Diazyme Co.).

### Statistical analysis

The data were analyzed by SPSS package Version 13. Statistical analysis was performed using the independent and paired samples t-test. Pearson correlation coefficient was also applied for correlation estimation between malondialdehyde and inflammatory markers. P-values were considered significant at <0.05.

### Virtual analysis and bioinformatics data

In silico experiment was performed in a virtual interactive environment using Molegro Virtual Docking Software Ver. 4.2. COX-2 X-ray crystallographic and ligand structures were obtained from protein data bank [Protein Data Bank (PDB) ID code 6COX] and Zinc Cheminformatics data base [24]. Docking method was energy minimization the results of which were obtained according to the MolDock scores. Bioinformatics data from protein data bank were also accessed. Cheminformatics structures were (+)-Catechin and (−)-Epicatechin composing the main ingredients of Cocoa.

## Results

### Clinical experiment results

**Figure 1:**
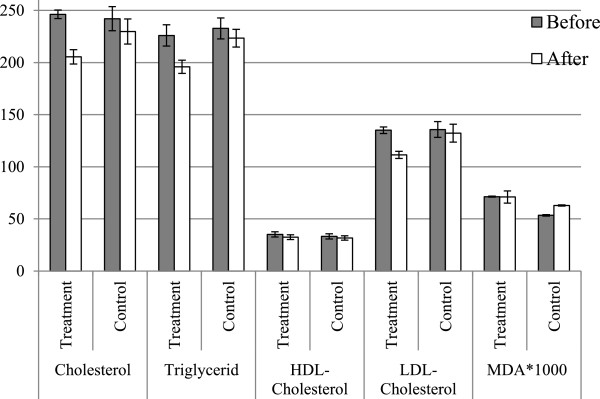
Comparaison of biochemical markers of lipid profile and lipid peroxidation between treatment and control categories.

The ratio of males to females was the same in both groups. The patient’s mean age was 54 ± 5 and the mean score for body mass index (BMI) was 28 ± 5 kg/m^2^. Table 1 illustrates the comparison of serum lipids, lipoproteins and malondialdehyde of the treatment and control groups before and after the intervention. Data analysis for comparison between the groups before and after a 6-week intervention (Figure 1) detects that total cholesterol, triglyceride, HDL-C, LDL-C and MDA are changed significantly only in TG: 16.57% total cholesterol reduction in TG, P < 0.0001 vs. 5.08% reduction in CG, P = 0.0006; 13.3% triglyceride reduction in TG, P < 0.0001 vs. 3.99% in CG, P = 0.0009; 7.58% reduction of HDL-C in TG, P = 0.0003 vs. 4.57% in CG, P = 0.0258; 17.54% reduction of LDL-C in TG, P < 0.0001 vs. 2.57% in CG, P = 0.1308; malondialdehyde levels, however, were not reduced significantly in TG (0.45%, P = 0.787) but increased significantly in CG (17.75%, P < 0.0001).

**Table 1:** Comparison of serum levels of lipids, lipoproteins and MDA in type 2 diabetic patients of treatment and control categories before and after intervention; Under treatment individuals were under Cocoa plus milk powder regimen but control individuals were only under milk powder regimen, both groups, for 6 weeks

**Biochemical indices**	**Categories**	**Before intervention mean ± SD**	**P-value**	**After intervention mean ± SD**	**P-value**
**Total cholesterol**	Treatment	246.22 ± 4.14	0.0172	205.42 ± 6.89	<0.0001
(mg/dl)	Control	242 ± 11.6		229.7 ± 11.98	
**Triglyceride**	Treatment	226 ± 10.15	0.0013	195.92 ± 6.30	<0.0001
(mg/dl)	Control	232.7 ± 10.04		223.4 ± 8.52	
**HDL-cholesterol**	Treatment	35.06 ± 2.53	0.0003	32.4 ± 2.25	0.1066
(mg/dl)	Control	33.2 ± 2.49		31.68 ± 2.17	
**LDL-cholesterol**	Treatment	135.08 ± 3.15	0.5818	111.38 ± 3.37	<0.0001
(mg/dl)	Control	135.72 ± 7.56		132.22 ± 8.51	
**MDAx1000**	Treatment	71.32 ± 0.55	<0.0001	71 ± 5.87	<0.0001
(nmol)	Control	53.4 ± 0.62		62.88 ± 0.54	

As presented in Table 2, after 6 weeks of Cocoa powder consumption, there were significant decreases for serum levels of hs-CRP, IL-6, and TNF-α respectively (CI = 0.95, P < 0.05). Moreover, the results revealed a positive correlation between malondialdehyde and inflammatory markers (hs-CRP, IL-6, TNF-α) which is statistically significant (P < 0.05) (Table 3).

**Table 2:** Blood level of inflammatory markers in type 2 diabetic patients before consumption of Cocoa plus milk and after 6 weeks of consumption twice daily

**Inflammatory markers**	**Before cocoa powder consumption (Mean ± SD)**	**After cocoa powder consomption (Mean ± SD)**	**P-value**
**hs-CRP (mg/dl)**	0.43 ± 0.32	0.23 ± 0.31	0.0294
**IL-6 (pg/ml)**	2.5 ± 0.9	1.7 ± 0.7	0.001
**TNF-α (pg/ml)**	2.64 ± 0.95	1.38 ± 0.28	<0.0001

**Table 3:** Correlation table of malondialdehyde and inflammatory markers hs-CRP, IL-6, TNF-α

**Inflammatory marker**	**Malondialdehyde (MDA)**	**P-value**
hs-CRP	0.090	<0.05
IL-6	0.076	<0.05
TNF-α	0.076	<0.05

### Bioinformatics data and virtual analysis results

**Figure 2:**
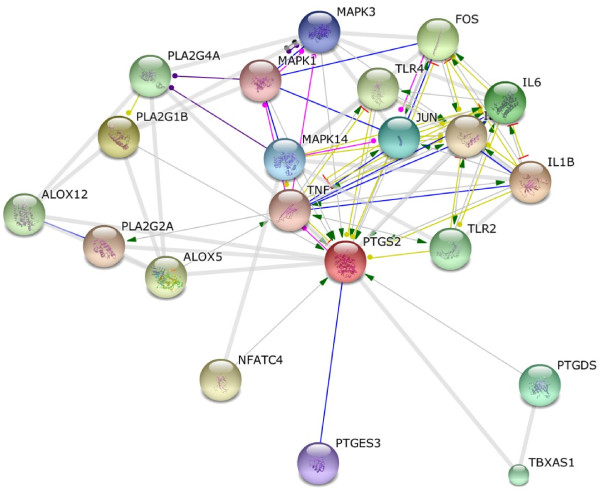
**Prostaglandine synthase-2 (PTGS-2) interactive network obtained from string data base.** The criteria for screening showed proteins were the score more than 0.98% relevancies.

**Figure 3:**
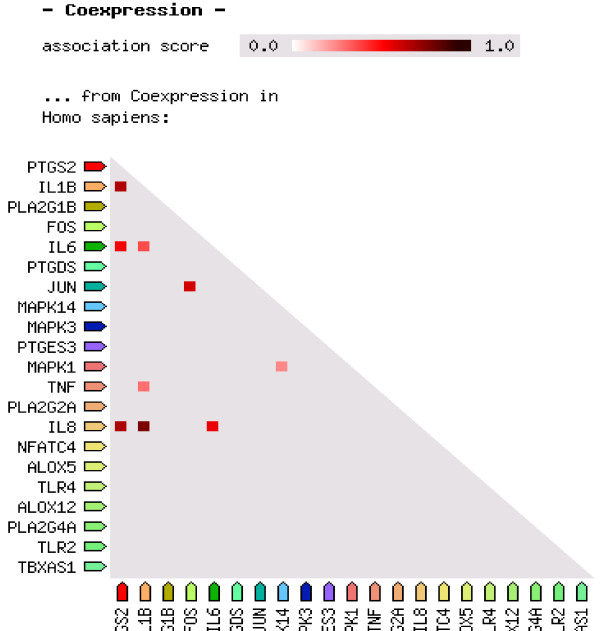
**This figure shows coexpression of 20 selected proteins.** PTGS-2 is mostly coexpressed concurrently with IL1B, IL8 and IL6.

**Figure 4:**
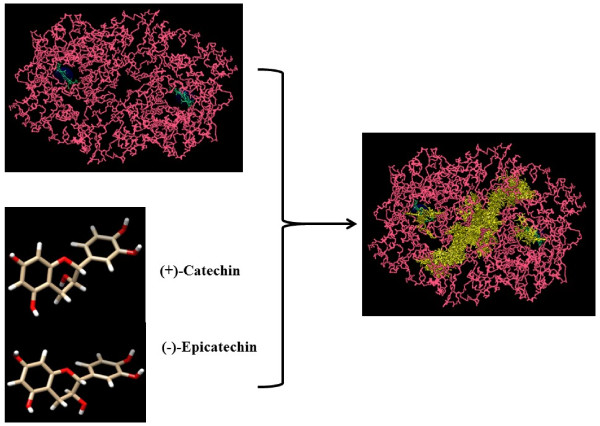
**Quaternary structure of PTGS-2 depicted in Molegro Virtual Docker software.** 2 Heme groups which are prosthetic groups are located in the active site of enzyme. Also structures of (+)-Catechin and (−)-Epicatechin are depicted in Chimera software; the atoms of Catechin and Epicatechin are shown by: oxygen in red color, hydrogen in white and the else are carbon.

Prostaglandin synthase-2 (PTGS-2/COX-2) belongs to the peroxidase protein family. According to the string data base there are more than 50 other proteins that have possibly no interaction with or effect on the COX-2/PTGS-2. However, an interactive network obtained from string data base suggests that PTGS-2 is affected by, or even exerts influence on Tumor Necrosis Factor alpha (TNF-α), Interlukin-6 (IL-6), Interlukin-1B (IL-1B), Toll Like receptors (TLR), prostaglandins, cell signaling mediators, activators and so forth. As it is shown in Figure 2, PTGS-2 is activated by TNF-α and IL6 pathways; both molecules were measured in our interventional clinical study. Figure 3 shows coexpression of PTGS-2 with proteins of Figure 2 network; this figure implies that PTGS-2 is mostly coexpressed concurrently with IL1B, IL8 and IL6. Therefore, for investigation of inflammation, increment of IL8 and IL1B may be obvious in addition to IL6. Figure 4 depicts an X-ray crystallographic structure of PTGS-2.

### Docking results

**Figure 5:**
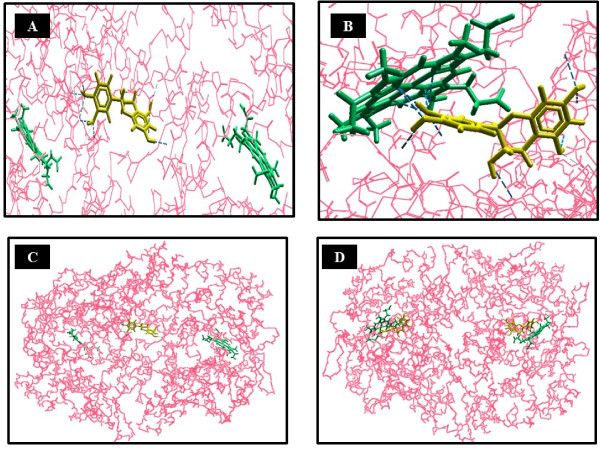
**Interaction of Cocoa most frequent ingredients Catechin and Epicatechin with PTGS-2/COX-2; A: depicts interaction of Catechin with PTGS-2 in the position with the highest score according to the MolDock score. B**: depicts the interaction Catechin/ Epicatechin with Heme prosthetic group of PTGS-2 (−131.715); **C**: depicts a rough shape of PTGS-2 interaction with Catechin molecule and its steric position; **D**: depicts Catechin (left) and Epicatechin (right) interaction with Heme prosthetic groups.

Docking studies of (+)-Catechin and (−)-Epicatechin with PTGS-2 resulted in good interactions, as schematically shown in the Figure 5, depicting the best sites of protein-ligand interaction sites; Catechin interacts with PTGS-2 in various positions but mostly with the interface domains of two chains. As the scoring criteria of docking is on the basis of MolDock score and is reported as representative of Gibbs free energy (delta-G), therefore, the more negative the mean score, the better interaction value and status. Interaction of ligand-acceptor is scored according to the MolDock score in Molegro Virtual Docker software. In the present in silico analysis, the best score is −135.315 for Catechin and in a position that ten hydrogen bounds (shown as blue color in Figure 5) among amino acids Tyrosine 373, Serine 121 and 126, Phenylalanine 367 and 2 × 371 (bifurcated hydrogen bounds), Lysine 532, Leucine 344, Isoleucine 124, and Glutamine 369. Another important and interesting interaction is one of Catechin/Epicatechin with Heme prosthetic group of PTGS-2 that is scored according to the MolDock score equal to −131.715 in the best status for Catechin interaction with Heme prosthetic group. Anyway, Epicatechin interacts with Heme by a score equal to −126.31. However, this latest noted interaction in fact is the best interaction of Epicatechin-PTGS-2.

## Discussion

The protective effect of dietary flavonoids against many diseases in particular cardiovascular disease and diabetes are supported by several studies [12,13,25-28]. Cocoa is a rich source of polyphenols. The flavan-3-ol monomers, catechin, epicatechin, and the oligomeric procyanidins are the major flavonoids in Cocoa [25]. Attention has been directed to the antioxidant potential of these flavonoids of Cocoa and their potentially protective effects against the risk of cardiovascular disease [17,25,26,29]. In the current study, our results have shown favorable effects on serum lipids and lipoprotein concentrations in type 2 diabetic patients after 6 weeks of Cocoa consumption. Also in this study we have addressed whether a 6-week consumption of Cocoa produces a beneficial effect on blood lipid peroxidation. Our results show that consumption of Cocoa significantly reduces serum levels of total cholesterol, triglyceride, HDL-C and LDL-C in type 2 diabetic patients after 6 weeks. Previous studies have reported that polyphenol-rich Cocoa and chocolate reduce LDL-C in humans [13,25]. Baba S. et al. detected that consumption of 26 grams/day of Cocoa powder for 12 weeks lowers plasma LDL-C by 11% in healthy men [12]. This report supports our finding on LDL-C. Other studies have also reported that polyphenol-rich dark chocolate and Cocoa powder increases plasma HDL-cholesterol [12,13,25-28]. Wan et al. have identified that after daily consumption of 22 g of Cocoa powder for 4 weeks, the concentration of HDL-cholesterol was 4% higher following a control diet [26]. These findings suggest that absorbed polyphenolic substances in Cocoa powder, such as Catechin and Epicatechin, may affect plasma HDL-C concentration. In our study, however, HDL-C concentrations decreased significantly in both treatment and control groups; such effect may be due to the changes of dietary regimen in study population and the effect of milk and Cocoa lipoprotein and lipid contents which may change the type of lipids of diabetic patients. Moreover, consumption of Cocoa has been found to inhibit lipid peroxidation in treatment group; malondialdehyde concentrations in type 2 diabetic patients who were on the Cocoa regimen did not change significantly but increased in patients bereft of Cocoa supplement. As some studies have shown that consumption of Cocoa decreases the formation of lipid oxidation products such as malondialdehyde [28,29], and in line with our results, we propose that such impact is more preventive than lowering. Decreased rates of lipid peroxidation in diabetic patients after Cocoa administration suggest that such effects may be due to flavonoids contents of Cocoa.

Inflammation plays a pivotal role in cardiovascular events. Studies suggest that lifestyle and drug interventions which reduce hs-CRP levels typically delay and decrease the risk of cardiovascular diseases [30,31]. However, there is a limited number of controlled study to define the effects of individual dietary constituents on biomarkers of inflammation [32]. Mathur et al. have shown no significant change in whole-blood cytokines, IL-6, TNF-α and hs-CRP after 6 weeks of Cocoa supplementation [33]. However, the results of our study revealed that serum level of TNF-α, IL-6 and hs-CRP decreases significantly in patients under Cocoa powder regimen. The correlation between malondialdehyde and inflammatory markers (hs-CRP, IL-6, TNF-α) indicated that a significant positive correlation exists between them. Considering such inflammatory markers, it could be suggested that Cocoa flavonoids represent an exciting new area of nutritional research especially as a cardiovascular damage protective agents in diabetic patients. Anyway, further experimental studies with Cocoa flavonoids are required to define the specific mechanisms of action on the inflammatory markers. Long-term studies with large sample sizes are also warranted to determine optimal doses and long-term effects of flavonoids, including Cocoa.

**Figure 6:**
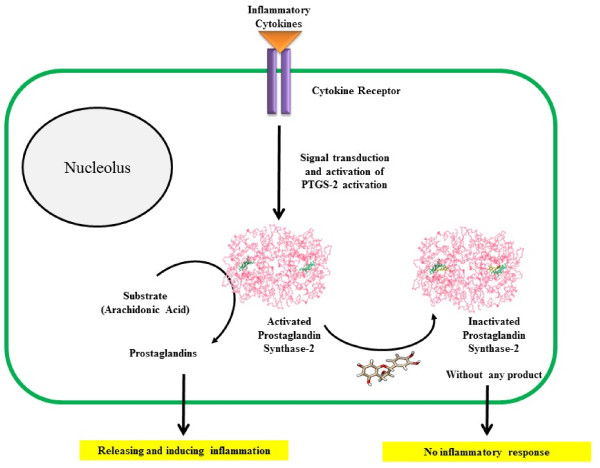
**Conceptual map depicted according to the virtual analysis and clinical results from our interventional study.** Inflammatory cytokines induce activation of PTGS-2 via signal transduction pathways.

However, bioinformatics and docking analysis were helpful for exploring some potential evidences of observed effects of Cocoa consumption which may be addressed as the following: a) As shown in Figure 2, PTGS-2 inflammatory enzyme is activated by TNF-α, IL6, IL1B, TLR and so forth, then inhibition of inflammation by Cocoa ingredients is probable especially via inhibition of PTGS-2, as is expected according to in silico/docking analysis which catalyses the production of inflammatory prostanoids. Therefore, inductive effect and responses of inflammatory cytokines such as TNF-α, IL6, IL1B, IL8 and prostaglandins all became obstructed by inhibition of PTGS-2; b) As represented in Figure 3, PTGS-2 has coexpression with IL8, IL1B and IL6. So it is likely that not only TNF-α and IL6 but also other more valuable agents involved in inflammation processes may exist. Different types of proteins, therefore, could be targeted by ligands such as Catechin and Epicatechin. In view of the fact that inflammation induces lipid peroxidation [34,35] and regarding that PTGS-2 has a main role in production of inflammatory prostanoids, it is proposed that decreased level of serum total cholesterol, Triglycerides, LDL-cholesterol, and inhibition of malondialdehyde formation after 6-week Cocoa consumption (Table 1) be all related to the interaction and probably inhibition of PTGS-2 by Catechin and Epicatechin. Such event, according to the virtual analysis, results in beneficial effects on lipid and lipoprotein metabolism and potentially via unknown ways. Furthermore, decreased levels of inflammatory markers TNF-α, IL6 and hs-CRP may also be related to the probable interaction of Catechin and Epicatechin with signaling molecules in the pathways inducing such markers production thus requiring more investigations. Another interesting finding of docking result is the interaction of Catechin/Epicatechin with prosthetic groups of PTGS-2. PTGS-2 has two groups of Heme. Heme is the prosthetic group and then a part of enzyme active site. Regarding the fact that binding of a ligand to the active site of an enzyme could halt catalytic activity, we propose that the interaction of Cocoa ingredients such as Catechin/Epicatechin with PTGS-2 is accomplished via Heme, which in turn, results in the inhibition of the enzyme, reduction of the inflammation and the manifestation of the results we have reported in our clinical experiments. Altogether, a conceptual diagram based on the virtual and clinical results can be proposed as in Figure 6. According to Figure 6 we thus can propose Cocoa or its active ingredients as anti-inflammatory agents that their mechanism of action, for example signaling molecules mediators, should be precisely described and unraveling aspects of this calls for further research.

## Competing interests

Authors declare that there is no conflict of interest for publishing this original study.

## Authors’ contributions

NP wrote the initial drafts. HMK, AA and MRM revised the manuscript. All authors read and approved the final manuscript.

## Authors’ information

NP and HMK are scientific members of Biochemistry and nutrition department, respectively AA is biochemistry ph.D student. They designed and supervised this study. MRM from English Language Department made a comprehensive revision of the article. All authors read and approved the final manuscript.

## References

[B1] Bonora E Kiechl S Willeit J Oberhollenzer F Egger G Targher G Alberiche M Bonadonna RC Muggeo M Prevalence of insulin resistance in metabolic disorders: the Bruneck study Diabetes 1998 47 10 1643 1649 10.2337/diabetes.47.10.1643 9753305

[B2] Hotamisligil GS Inflammation and metabolic disorders Nature 2006 444 7121 860 867 10.1038/nature05485 17167474

[B3] Pradhan A Obesity, metabolic syndrome, and type 2 diabetes: inflammatory basis of glucose metabolic disorders Nutr Rev 2007 65 s3 S152 S156 18240540 10.1111/j.1753-4887.2007.tb00354.x

[B4] Menotti A Kromhout D Blackburn H Fidanza F Buzina R Nissinen A Food intake patterns and 25-year mortality from coronary heart disease: cross-cultural correlations in the seven countries study Eur J Epidemiol 1999 15 6 507 515 10.1023/A:1007529206050 10485342

[B5] Ceriello A Motz E Is oxidative stress the pathogenic mechanism underlying insulin resistance, diabetes, and cardiovascular disease? The common soil hypothesis revisited Arterioscler Thromb Vasc Biol 2004 24 5 816 823 10.1161/01.ATV.0000122852.22604.78 14976002

[B6] Walter MF Jacob RF Jeffers B Ghadanfar MM Preston GM Buch J Mason RP Serum levels of thiobarbituric acid reactive substances predict cardiovascular events in patients with stable coronary artery disease: a longitudinal analysis of the PREVENT study J Am Coll Cardiol 2004 44 10 1996 2002 10.1016/j.jacc.2004.08.029 15542282

[B7] Dandona P Aljada A Chaudhuri A Bandyopadhyay A The potential influence of inflammation and insulin resistance on the pathogenesis and treatment of atherosclerosis-related complications in type 2 diabetes J Clin Endocrinol Metab 2003 88 6 2422 2429 10.1210/jc.2003-030178 12788837

[B8] Libby P Ridker PM Maseri A Inflammation and atherosclerosis Circulation 2002 105 9 1135 1143 10.1161/hc0902.104353 11877368

[B9] Du Clos TW Function of C-reactive protein Ann Med 2000 32 4 274 278 10.3109/07853890009011772 10852144

[B10] Voutilainen S Nurmi T Mursu J Rissanen TH Carotenoids and cardiovascular health Am J Clin Nutr 2006 83 6 1265 1271 16762935 10.1093/ajcn/83.6.1265

[B11] Natsume M Osakabe N Yamagishi M Takizawa T Nakamura T Miyatake H Hatano T Yoshida Tb Analyses of polyphenols in cacao liquor, cocoa, and chocolate by normal-phase and reversed-phase HPLC Biosci Biotechnol Biochem 2000 64 12 2581 2587 10.1271/bbb.64.2581 11210120

[B12] Baba S Osakabe N Natsume M Long-term intake of cocoa powder reduce the oxidative susceptibility of low density lipoprotein in healthy humans.(in Japanese) Jpn J Med Pharm Sci 2004 52 947 963

[B13] Baba S Osakabe N Kato Y Natsume M Yasuda A Kido T Fukuda K Muto Y Kondo K Continuous intake of polyphenolic compounds containing cocoa powder reduces LDL oxidative susceptibility and has beneficial effects on plasma HDL-cholesterol concentrations in humans Am J Clin Nutr 2007 85 3 709 717 17344491 10.1093/ajcn/85.3.709

[B14] Monagas M Khan N Andrés-Lacueva C Urpí-Sardá M Vázquez-Agell M Lamuela-Raventós RM Estruch R Dihydroxylated phenolic acids derived from microbial metabolism reduce lipopolysaccharide-stimulated cytokine secretion by human peripheral blood mononuclear cells Br J Nutr 2009 102 02 201 206 10.1017/S0007114508162110 19586571

[B15] Hla T Neilson K Human cyclooxygenase-2 cDNA Proc Natl Acad Sci 1992 89 16 7384 7388 10.1073/pnas.89.16.7384 1380156 PMC49714

[B16] Kurumbail RG Stevens AM Gierse JK McDonald JJ Stegeman RA Pak JY Gildehaus D Miyashiro JM Penning TD Seibert K Isakson PC Stallings WC Structural basis for selective inhibition of cyclooxygenase-2 by anti-inflammatory agents Nature 1996 384 6610 644 648 10.1038/384644a0 8967954

[B17] Kris-Ethertona PM Keenb CL Evidence that the antioxidant flavonoids in tea and cocoa are beneficial for cardiovascular health Curr Opin Lipidol 2002 13 41 49 10.1097/00041433-200202000-00007 11790962

[B18] Waterhouse AL Shirley JR Donovan JL Antioxidants in chocolate Lancet 1996 348 9030 834 8814019 10.1016/S0140-6736(05)65262-2

[B19] Sanbongi C Osakabe N Natsume M Takizawa T Gomi S Osawa T Antioxidative polyphenols isolated from Theobroma cacao J Agric Food Chem 1998 46 2 454 457 10.1021/jf970575o 10554262

[B20] Mao T Powell J Van de Water J Keen C Schmitz H Gershwin M Effect of cocoa procyanidins on the secretion of interleukin-4 in peripheral blood mononuclear cells J Med Food 2000 3 2 107 114 10.1089/109662000416294

[B21] Mao T Van de Water J Keen C Schmitz H Gershwin M Modulation of TNF-α secretion in peripheral blood mononuclear cells by cocoa flavanols and procyanidins Clin Dev Immunol 2002 9 3 135 141 10.1080/1044667031000137601 PMC2276101 12885154

[B22] Friedewald WT Levy RI Fredrickson DS Estimation of the concentration of low-density lipoprotein cholesterol in plasma, without use of the preparative ultracentrifuge Clin Chem 1972 18 6 499 502 4337382

[B23] Hodges DM DeLong JM Forney CF Prange RK Improving the thiobarbituric acid-reactive-substances assay for estimating lipid peroxidation in plant tissues containing anthocyanin and other interfering compounds Planta 1999 207 4 604 611 10.1007/s004250050524 28456836

[B24] Irwin JJ Shoichet BK ZINC–a free database of commercially available compounds for virtual screening J Chem Inf Model 2005 45 177 182 10.1021/ci049714+ 15667143 PMC1360656

[B25] Keen CL Holt RR Oteiza PI Fraga CG Schmitz HH Cocoa antioxidants and cardiovascular health Am J Clin Nutr 2005 81 1 298S 303S 15640494 10.1093/ajcn/81.1.298S

[B26] Wan Y Vinson JA Etherton TD Proch J Lazarus SA Kris-Etherton PM Effects of cocoa powder and dark chocolate on LDL oxidative susceptibility and prostaglandin concentrations in humans Am J Clin Nutr 2001 74 5 596 602 11684527 10.1093/ajcn/74.5.596

[B27] Safeer RS Cornell MO The emerging role of HDL cholesterol Postgrad Med 2000 108 7 87 90 11126145 10.3810/pgm.2000.12.1319

[B28] Osakabe N Baba S Yasuda A Iwamoto T Kamiyama M Takizawa T Itakura H Kondo K Daily cocoa intake reduces the susceptibility of low-density lipoprotein to oxidation as demonstrated in healthy human volunteers Free Radic Res 2001 34 1 93 99 10.1080/10715760100300091 11235000

[B29] Erdman JW Jr Carson L Kwik-Uribe C Evans EM Allen RR Effects of cocoa flavanols on risk factors for cardiovascular disease Asia Pac J Clin Nutr 2008 17 Suppl 1 284 287 18296357

[B30] Kleemann R Verschuren L de Rooij BJ Lindeman J de Maat MM Szalai AJ Princen HMG Kooistra T Evidence for anti-inflammatory activity of statins and PPARα activators in human C-reactive protein transgenic mice in vivo and in cultured human hepatocytes in vitro Blood 2004 103 11 4188 4194 10.1182/blood-2003-11-3791 14976045

[B31] Ridker PM Danielson E Fonseca F Genest J Gotto AM Jr Kastelein J Koenig W Libby P Lorenzatti AJ MacFadyen JG Rosuvastatin to prevent vascular events in men and women with elevated C-reactive protein N Engl J Med 2008 359 21 2195 10.1056/NEJMoa0807646 18997196

[B32] Basu A Devaraj S Jialal I Dietary factors that promote or retard inflammation Arterioscler Thromb Vasc Biol 2006 26 5 995 1001 10.1161/01.ATV.0000214295.86079.d1 16484595

[B33] Mathur S Devaraj S Grundy SM Jialal I Cocoa products decrease low density lipoprotein oxidative susceptibility but do not affect biomarkers of inflammation in humans J Nutr 2002 132 12 3663 3667 12468604 10.1093/jn/132.12.3663

[B34] Memon RA Staprans I Noor M Holleran WM Uchida Y Moser AH Feingold KR Grunfeld C Infection and inflammation induce LDL oxidation in vivo Arterioscler Thromb Vasc Biol 2000 20 6 1536 1542 10.1161/01.ATV.20.6.1536 10845869

[B35] Casini A Ceni E Salzano R Biondi P Parola M Galli A Foschi M Caligiuri A Pinzani M Surrenti C Neutrophil‐derived superoxide anion induces lipid peroxidation and stimulates collagen synthesis in human hepatic stellate cells: role of nitric oxide Hepatology 2003 25 2 361 367 9021948 10.1053/jhep.1997.v25.pm0009021948

